# Beneficial Effects of Tamarind Trypsin Inhibitor in Chitosan–Whey Protein Nanoparticles on Hepatic Injury Induced High Glycemic Index Diet: A Preclinical Study

**DOI:** 10.3390/ijms22189968

**Published:** 2021-09-15

**Authors:** Ana J. F. C. Aguiar, Jaluza L. C. de Queiroz, Pedro P. A. Santos, Christina S. Camillo, Alexandre C. Serquiz, Izael S. Costa, Gerciane S. Oliveira, Ana F. T. Gomes, Lídia L. R. Matias, Rafael O. A. Costa, Thaís S. Passos, Ana H. A. Morais

**Affiliations:** 1 Biochemistry and Molecular Biology Postgraduate Program, Biosciences Center, Federal University of Rio Grande do Norte, Natal 59.078-970, RN, Brazil; anajulianutri@hotmail.com (A.J.F.C.A.); luh_nutri@hotmail.com (J.L.C.d.Q.); izaeldesousa91@gmail.com (I.S.C.); lidialeonize@gmail.com (L.L.R.M.); rafaeloliveira.nutri@gmail.com (R.O.A.C.); 2 Structural and Functional Biology Postgraduate Program, Biosciences Center, Federal University of Rio Grande do Norte, Natal 59.078-970, RN, Brazil; ppdasantos@gmail.com (P.P.A.S.); camillosc@hotmail.com (C.S.C.); 3 Nutrition Course, University Center of Rio Grande do Norte, Natal 59.014-545, RN, Brazil; alexandreserquiz@gmail.com; 4 Nutrition Course, Potiguar University, Natal 59.056-000, RN, Brazil; 5 Nutrition Postgraduate Program, Center for Health Sciences, Federal University of Rio Grande do Norte, Natal 59.078-970, RN, Brazil; gercianesilva2@hotmail.com (G.S.O.); aft.gomes00@gmail.com (A.F.T.G.); 6 Department of Nutrition, Center for Health Sciences, Federal University of Rio Grande do Norte, Natal 59.078-970, RN, Brazil; thais_spassos@yahoo.com.br

**Keywords:** nanoencapsulation, protease inhibitor, hyperglycemia

## Abstract

Several studies have sought new therapies for obesity and liver diseases. This study investigated the effect of the trypsin inhibitor isolated from tamarind seeds (TTI), nanoencapsulated in chitosan and whey protein isolate (ECW), on the liver health status of the Wistar rats fed with a high glycemic index (HGLI) diet. The nanoformulations without TTI (CW) and ECW were obtained by nanoprecipitation technique, physically and chemically characterized, and then administered to the animals. The adult male Wistar rats (*n* = 20) were allocated to four groups: HGLI diet + water; standard diet + water; HGLI diet + ECW (12.5 mg/kg); and HGLI diet + CW (10.0 mg/kg), 1 mL per gagave, for ten days. They were evaluated using biochemical and hematological parameters, Fibrosis-4 Index for Liver Fibrosis (FIB-4), AST to Platelet Ratio Index (APRI) scores, and liver morphology. Both nanoparticles presented spherical shape, smooth surface, and nanometric size [120.7 nm (ECW) and 136.4 nm (CW)]. In animals, ECW reduced (*p* < 0.05) blood glucose (17%), glutamic oxalacetic transaminase (39%), and alkaline phosphatase (24%). Besides, ECW reduced (*p* < 0.05) APRI and FIB-4 scores and presented a better aspect of hepatic morphology. ECW promoted benefits over a liver injury caused by the HGLI diet.

## 1. Introduction

In recent decades, there has been a nutritional transition worldwide with changes in people’s eating habits of all age groups, which has been a demonstrated preference for ultra-processed foods. Among these products are high glycemic index and/or high glycemic load foods, which have high palatability and low cost, including fast foods, drinks with added sugar, soft drinks, snacks, and sweets [1,2,3]. In addition, a sedentary lifestyle associated with poor quality of food consumption leads to a higher prevalence of chronic non-communicable diseases (NCDs), such as obesity [4].

The growing and alarming increase in the prevalence of obesity is a public health problem worldwide, affecting the quality and life expectancy of the population. In addition, it causes an overload on health systems since excess fat is associated with several comorbidities, including metabolic syndrome (MS), cancer, cardiovascular diseases, hypertension, type 2 diabetes mellitus (DM2), liver diseases, and others [5,6,7]. For example, among liver diseases, Non-Alcoholic Fatty Liver Disease (NAFLD) is currently one of the causes of chronic liver disease, with an estimated global prevalence of 25–30%, increasing up to 90% in patients with morbid obesity. Therefore, it is increasingly recognized as the component of liver disease in MS [8,9].

NAFLD is defined as the presence of ≥5% hepatic steatosis (HE) in the absence of competitive liver disease etiologies, such as chronic viral hepatitis, use of drugs that induce steatosis, and other chronic liver diseases, in addition to not having significant alcohol consumption. In addition, NAFLD includes steatosis to non-alcoholic steatohepatitis (NASH) that can progress to cirrhosis and hepatocellular carcinoma [8,9,10,11].

With the increasing prevalence of NAFLD and clinical severity outcomes, there is a growing need for a robust, precise, and non-invasive approach to diagnosing the different stages of this condition, such as analysis of liver biochemical parameters, imaging tests, or risk screening [12]. Global guidelines agree that the ideal strategy for stratifying NAFLD patients and tracking disease progression has not yet been established. According to current guidelines, a combination of assessing biochemical parameters, non-invasive scores, and imaging tests to identify patients at low risk for advanced liver disease and clinical decision making should be used [12,13,14,15]. In addition, this combination can identify patients who must undergo a liver biopsy to confirm advanced fibrosis and in which a more intensive approach is required [16,17].

NAFLD pharmacotherapy is an unmet medical need, so basic and translational research on NAFLD animal models is indispensable. Although the number of studies has increased and animal models are currently used, there is an ongoing challenge to identify the models that reflect the closest human form of presentation to allow a good translation of the results obtained in later clinical development [18]. In addition, diets rich in carbohydrates more relative to the current human dietary intake have already been shown to affect NAFLD in animal models, for example, the high glycemic index and high glycemic load (HGLI) diet [19,20].

Because it is a highly prevalent disease in the Western world, with complicated differential diagnoses and the absence of specific drugs, researchers must seek to study possible molecules, bioactive substances, and/or drugs to treat and prevent disease progression. In this context, protease inhibitors have aroused interest, as reports point to different applications in the health area [21,22].

Several published studies have evaluated a Kunitz-type tamarind seed trypsin inhibitor (TTI) in experimental models. They have pointed out effects, such as reduced food consumption in obese [23,24] and eutrophic [25] Wistar rats, along with the improvement of the lipid profile [reducing very-low-density lipoprotein (VLDL-c) and triglycerides] [26], the inflammatory profile reduction of Tumor Necrosis Factors α -TNF-α) [24], and decrease of leptin regardless of body weight [23,27].

Recently, new studies have been developed on the bioactive effects of a nanoformulation based on TTI isolate, chitosan, and whey protein isolate (ECW), which has a spherical shape, smooth surface, size close to 100 nm, and potentiation of antitrypsin activity of the TTI [28]. In addition, ECW presented safe in vitro and in vivo toxicological evaluation [29] and induced a significant reduction in glutamic oxalacetic transaminase (AST) in an experiment with Wistar rats fed the HGLI diet mentioned above. In a third study with the same experimental model, ECW demonstrated a hypoglycemic effect in vivo [30]. Thus, it is noteworthy that both the impact on the significant reduction in AST and fasting glucose had not yet been attributed to isolated, non-encapsulated TTI. Therefore, further studies are needed to assess whether nanoformulation can be promising for use in NAFLD treatment due to the effects directly related to encapsulation.

In this context, further research must be carried out to investigate the pathogenesis of the disease in experimental models with dietary patterns similar to the western diet (with a high carbohydrate content). Besides, since management and therapy, evaluating new therapeutic interventions is necessary for public health [12,14,15]. Therefore, this study aims to assess the effect of the trypsin inhibitor isolated from the tamarind seed (*Tamarindus indica* L.) nanoencapsulated on hematological and biochemical parameters, liver damage assessment scores, and histopathology of Wistar rat livers fed the HGLI diet.

## 2. Results and Discussion

### 2.1. Obtainment and Characterization of TTI and Nanoformulations (CW and ECW)

**Figure 1:**
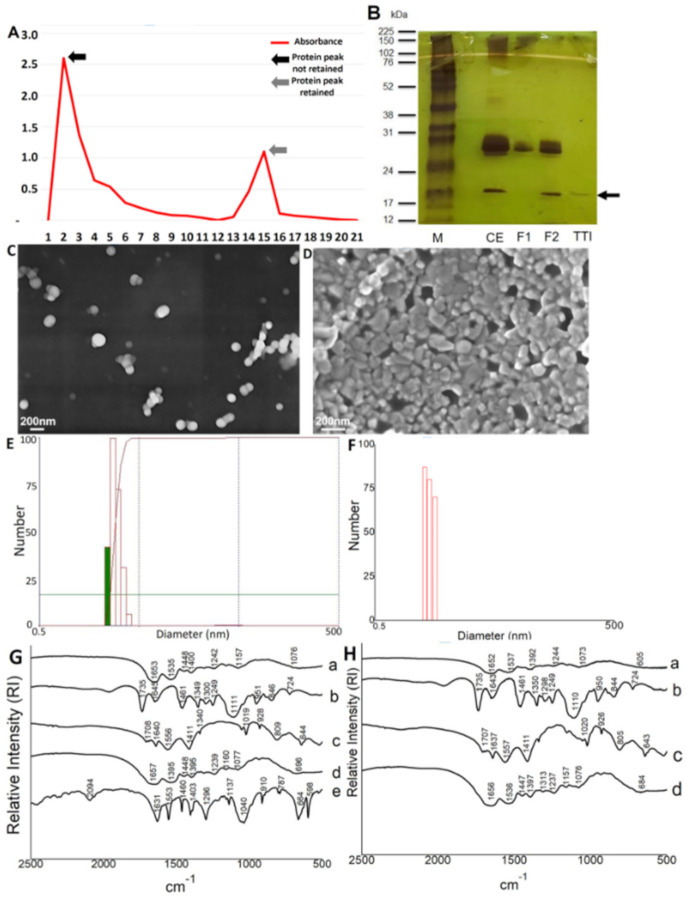
Isolation of TTI and characterization of nanoformulations (ECW and CW). (**A**) Chromatographic profile of F2 (which showed the highest antitrypsin activity) of tamarind seeds evaluated by spectrophotometry (Ultrospec™ 2100 pro UV/Visible Spectrophotometer, GE Heathcare Bio-Sciences Corp., Piscataway, NJ) at 280 nm. F2 = protein fraction 2. (**B**) Electrophoresis in denaturing polyacrylamide gel (SDS-PAGE) at 12.5% stained with silver nitrate. M = Marker; CE = Crude Extract; F1 = Protein fraction 1 (saturation with 0–30% ammonium sulfate); F2 = Protein fraction 2 (saturation with 30–60% ammonium sulfate); TTI = Trypsin inhibitor isolated from tamarind seeds after Trypsin-Sepharose affinity chromatography. (**C**) Scanning Electron Microscopy (SEM) of the ECW with a magnitude of 30 kX. (**D**) Scanning Electron Microscopy (SEM) of the CW with a magnification of 50 kX. (**E**) Laser diffraction obtained for ECW by dispersing nanoparticles in water and previous crosslinking using formaldehyde. (**F**) Laser diffraction obtained by CW by dispersing nanoparticles in water and previous crosslinking using formaldehyde. (**G**) ECW Fourier Transform Infrared Spectrum (FTIR): a. ECW; b. Tween 80; c. purified chitosan; d. whey protein isolate; e. TTI. (**H**) CW Fourier Transform Infrared Spectrum (FTIR): a. CW; b. Tween 80; c. purified chitosan; d. whey protein isolate. ECW trypsin inhibitor isolated from tamarind seeds encapsulated with whey protein isolate and chitosan (1:2:2 *w/w/w*); CW: chitosan–whey protein isolate nanoparticles (2:2 *w*/*w*).

In the chromatographic profile of F2 (which showed the highest antitrypsin activity) of tamarind seeds (Figure 1A), it is possible to observe the first peak of proteins with low-affinity trypsin. On the other hand, the second protein peak represents the TTI with high activity to inhibit trypsin, confirmed from the antitrypsin activity. After isolating the TTI by chromatography, 1.4 mg of TTI promoted 100% inhibition with specific antitrypsin activity of 1224 IU/mg.

The 12.5% SDS-PAGE gel stained with silver nitrate (Figure 1B) confirms the isolation of TTI with the presence of protein bands with a predominance of molecular mass around 20 kDa. The isolation and characterization of trypsin inhibitors from tamarind seeds have been well reported in the literature [23,24,25,26,27,29,30]. Therefore, the biochemical characteristics obtained in this study corroborate with previous studies.

After TTI encapsulation with whey protein isolate and chitosan by nanoprecipitation technique, the nanoformulations, with TTI (ECW) and without TTI (CW), were characterized by SEM, laser diffraction, and FTIR. It is worth mentioning that the CW nanoformulation was characterized to guarantee the administration of a nanoformulation with chemical and physical characteristics similar to the ECW. Thus, the results observed for the two nanoformulations could be compared. The obtained micrographs (Figure 1C,D) showed that both nanoencapsulated presented intact particles, spherical shape, smooth surface, and nanometric physical size. These results are in agreement with those found by Queiroz et al. [28], Matias et al. [30], and Costa et al. [29].

In the Laser Diffraction evaluation (Figure 1E,F), in aqueous media, the average diameter and polydispersity index were, respectively, 120.7 (5.31) nm and 0.475 (0.01) nm for ECW (Figure 1E) and 136.4 (22.35) nm and 0.29 (0.09) for CW (Figure 1F). These results prove the obtaining of nanometric particles with homogeneous size distribution [31]. For ECW, these results are close to those obtained by Matias et al. (117.4 nm; 0.37) [30] and Costa et al. (118 nm; 0.373) [29]. For the CW, it was observed that the results obtained were moderately superior when compared to those obtained by Matias et al. (123.9 nm; 0.23) [30].

FTIR analysis was performed to characterize the encapsulated products based on structure and chemical identification (Figure 1G,H). ECW spectrum (Figure 1G) showed bands corresponding to the TTI [1460 cm^−1^ and 1403 cm^−1^ (OH)] attenuated and displaced (1448 cm^−1^ and 1400 cm^−1^), indicating that the TTI was protected by encapsulating agents. Specifically, the band 1653 cm^−1^ (CO) in ECW (Figure 1G) and 1652 cm^−1^ (CO) in CW (Figure 1H) confirm the presence of whey protein isolate due to the observation of the vibrational stretching of amide I.

In addition, the presence of a new vibrational band formed in the region of 1076 cm^−1^ (C–O) was observed in the ECW (Figure 1G), which shows new chemical interactions formed between the encapsulating agents and the nucleus present in the nanoparticles. ECW and CW (Figure 1G,H) showed vibrational patterns of encapsulating agents used and new chemical bonds, displacement, and attenuation of the bands. In addition, the ECW also showed the presence of bands that confirmed the presence of TTI, displacements, and a new vibrational band, which characterized the interaction of TTI with the wall materials, indicating the encapsulation.

The encapsulation efficiency obtained in this study was 95.6% (0.64) for ECW. It is important to emphasize that the encapsulating agents and the CW nanoformulation did not show antitrypsin activity. These results revealed a high capacity for incorporating TTI into the particles while preserving functionality. The encapsulation efficiency is considered excellent when above 80% [32], as was observed in the present study. The result confirms strong chemical interactions in this system, showed in FTIR, mainly those of the polysaccharide-protein complex [33]. The results obtained corroborate with Queiroz et al. [28], Matias et al. [30], and Costa et al. [29].

It is important to highlight that these excellent results for nanoformulations, such as spherical, smooth particles, in nanometric and homogeneous size, with specific chemical interactions, indicate standardization and reproducibility of the production process. Therefore, it is essential to maintain the properties achieved through nanoencapsulation. Thus, even with the production of new lots of ECW and CW, the same physical and chemical characteristics presented by Costa et al. [29] and Matias et al. [30] have been observed.

### 2.2. Evaluation of Biochemical Parameters

After ten days of treatment with ECW, the hematological and biochemical parameters evaluated (Table 1) were hematological (hemoglobin, hematocrit, total leukocyte, and platelet count), biochemical (fasting glucose, insulin, HOMA-IR, HOMA-β), lipid profile (total cholesterol, LDL-c, HDL -c, VLDL-c, triglycerides), parameters for assessing renal function (urea, creatinine, total proteins, and albumin) and the assessing liver function (AST, ALT, GGT, and ALP).

#### 2.2.1. Hematological Parameters

Regarding the assessment of hematological parameters (Table 1), hemoglobin and hematocrit did not show a significant difference between groups (*p* > 0.05). This result is in line with Costa et al. [29], which found no significant difference for these two parameters, attesting to the low toxicity in vitro and in vivo and the safety of a possible clinical application of the nanoformulation ECW. Furthermore, another study carried out by Montero [34] in eutrophic Wistar rats, the effects of nanoformulations containing BSA and chitosan, also showed hemoglobin concentrations (12.48–16.03 g/dL) within the range observed in the present study (12.94–14.73 g/dL). It is important to highlight these similarities since changes in hematological parameters may be associated with some liver problems when evaluated in conjunction with other indicators of liver damage [12].

The leukocyte count showed a significant reduction (*p* = 0.006) in the group treated with CW compared to the other groups. Unfortunately, no studies were found that evaluated this parameter under the conditions proposed in the present study for CW. Matias et al. [30] reported on CW reactivity and the smaller particle size, which can be a likely explanation for this effect, but their study did not evaluate hematological parameters. However, it is already known that ECW does not cause this leukopenic effect [32], suggesting that TTI, when contained in ECW, was essential to normalize this evaluated parameter, probably because TTI reduces the reactivity of CW when interacting strongly with chitosan. Matias et al. [30] and Costa et al. [29] also reported the positive effect of TTI when contained in this nanoformulation (ECW). This data is important since NAFLD in advanced cases can cause low-grade inflammation, recruit inflammatory cells to the liver, and increase the recruitment of immune cells [8,12].

The platelet count showed no significant difference between the study groups (*p* > 0.05). This result was different from that obtained by Costa et al. [29], who observed a significant increase in platelet concentration (*p* < 0.05) for the group of animals treated with ECW. This increase is believed to be positive for the liver since thrombocytopenia is frequently seen in patients with chronic liver disease and cirrhosis. Although the relationship between thrombocytopenia, hepatic pathogenesis, and the role of platelets in chronic liver disease is poorly understood, some experimental evidence of platelets improving liver fibrosis and accelerating liver regeneration has been studied [35,36].

#### 2.2.2. Fasting Glucose, Insulin, and Model Homeostasis

The animals’ fasting glucose showed a significant difference (*p* = 0.028) between the group without treatment and the other groups (Table 1). The observed reduction was expected because the three groups underwent some treatment, either the conventional (standard diet) or nanoformulations (ECW and CW), promoting this reduction. Matias et al. [30] showed that this same effect had already been reported for animals treated with ECW and CW. The decrease in fasting glucose concentrations in the group that received CW is probably attributed to chitosan and whey protein isolate. The first is due to its hypoglycemic activity, already observed in previous studies [37,38], and the latter is related to effects on postprandial glucose homeostasis [39].

The hypoglycemic activity of ECW, which had already been reported by Matias et al. [30], is a new feature attributed to TTI since it had not been observed in a study with TTI isolate [24]. Regarding the liver, Chen et al. [40] observed that diabetic patients were more likely to have NAFLD. Concerning the relationship between carbohydrate metabolism, IR, and NAFLD, a significant reduction in fasting glucose in animals that received ECW shows improved glucose homeostasis, even with the continued consumption of a high glycemic index and high glycemic load diet.

Different from the benefits attributed to ECW, insulin, HOMA-IR, and HOMA-β showed a significant increase (*p* < 0.0001) in the group treated with CW compared to the other groups (Table 1). The other groups are within the parameters of normality described in Table 2. The Latin American Association for the Study of the Liver, in a practical guideline for the NAFLD diagnosis and treatment [12], assumes that the HOMA-IR value greater than or equal to 2.5 and hyperinsulinemia are some of the NAFLD diagnostic criteria.

The increased value in the groups that received the CW, both for insulin and HOMA values, may support the hypothesis that these animals had a worsening IR caused by the HGLI diet (according to HOMA-IR) and possibly close to dysfunction of β-pancreatic cells. Since HOMA-β measures this functionality and values greater than 100 indicate dysfunction, animals treated with CW obtained a HOMA-β value of 81.30. This fact has already been seen by Matias et al. [30]. Furthermore, they found from the histology of animals’ pancreas that those who received CW showed intense degeneration, hyperplasia of the pancreatic islets, and morphological changes in the pancreatic ducts. One more indication that the presence of TTI in the nanoformulation benefits the system and promotes positive effects.

Besides, Matias et al. [30] observed that the group treated with ECW presented a better morphological aspect of the pancreas. In the present study, the group of animals treated with ECW also showed a significant reduction (*p* > 0.05) in fasting glucose, suggesting a possible improvement in carbohydrate metabolism. Therefore, consequently minimizing the effect of IR in this group, raising the possibility that ECW could function as an adjuvant candidate in treating hyperglycemia and consequent NAFLD.

IR impairs glucose absorption by cells, resulting in a compensatory increase in insulin production by β cells. In addition to hyperinsulinemia, this progression of IR and problems with insulin production can lead to MS, pancreatic damage, dyslipidemia, and NAFLD [41]. Furthermore, the hypoglycemic action of ECW can be justified considering the hypothesis of Costa et al. [23]. They suggested that TTI might act as an insulin-like hormone, binding to specific receptors since ECW treatment did not influence insulin concentration.

#### 2.2.3. Lipid Profile

For the lipid profile of the animals, total cholesterol, triglycerides, HDL-c, LDL-c, and VLDL-c were evaluated (Table 1). For triglycerides, no significant difference was observed between groups (*p* > 0.05). There is no change in triglycerides and VLDL-c concentrations in the blood, possibly related to fat storage through triacylglycerol in the liver and not necessarily transport [42]. Evidence in the literature showed that the relationship between the content of intrahepatic triglycerides and the secretion rate of VLDL-c is curvilinear. VLDL-c exports increase linearly with the triglyceride content within the liver in individuals with normal intrahepatic triglyceride concentration. On the other hand, VLDL-c secretion reaches a plateau regardless of intrahepatic triglycerides in individuals with steatosis [43].

The concentrations of HDL-c (*p* = 0.0002) and total cholesterol (*p* = 0.0298) showed a significant reduction in the group treated with CW compared to the others. The decrease in HDL-c promoted by CW has already been observed by Matias et al. [30], which points to the beneficial effects sustained by ECW, attributed to the presence of TTI. This result was confirmed because, in peripheral tissues, HDL-c transports cholesterol in reverse. It transports this peripheral cholesterol to the liver, being discarded in bile. Thus, this reduction may be an indication of a worsening in lipid metabolism, in addition to being an anti-atherosclerotic molecule [44].

The reduction in total cholesterol in the group that received CW was precisely due to the decrease in HDL-c. On the other hand, the group treated with ECW was the highest average HDL-c concentration, explaining the non-reduction in total cholesterol. Thus, the harmful effects that the HGLI diet promotes in the liver, such as fat infiltration and dyslipidemia [19,20], seem to be enhanced by treatment with CW.

For LDL-c, no significant difference was observed between groups (*p* > 0.05). Therefore, it is already known that NAFLD is characterized by atherogenic dyslipidemia, postprandial lipemia, and HDL-c dysfunction. Most importantly, NAFLD patients have an increased risk of morbidity and mortality from the liver and cardiovascular disease (CVD). Therefore, assessing the lipid profile is essential to understand liver disease’s pathogenesis, diagnosis, and progression [45,46]. Although the group of animals treated with ECW did not show significant improvements in the concentrations of the lipid profile parameters, CW proved to be a disadvantageous treatment compared to the results obtained for ECW.

#### 2.2.4. Assessing Renal Function

Investigating the parameters for assessing renal impairment (Table 1) is essential in NAFLD since lipotoxicity in hepatic steatosis, caused by triglyceride accumulation in the tissues, also leads to IR [47], a risk factor for renal disease. In addition, NAFLD is a potential predictor of diabetic nephropathy, one of the main microvascular complications of DM2 [48]. Research conducted in diabetic and non-diabetic individuals has demonstrated the association between NAFLD and chronic kidney disease (CKD) [49,50,51].

Thus, creatinine and total proteins showed no significant difference between groups (*p* > 0.05). A similar result was found by Costa et al. [29] for total proteins, which analyzed ECW renal toxicity. Other studies with nanoparticles such as Feng et al. [52] and Tiwari et al. [53] obtained values close to creatinine observed in the present study (0.60–0.88 mg/dL).

There was a significant increase (*p* = 0.0015) in the urea concentration in the group treated with CW compared to the other groups. Conversely, there was a significant reduction (*p* < 0.001) in the group treated with CW compared to the other three groups for albumin. Costa et al. [29] also did not observe significant differences in urea and albumin concentrations in the animals treated with ECW compared to the other treatments and did not evaluate the group treated with CW. The decreased albumin levels may suggest a reduction in protein synthesis and/or renal dysfunction involved in protein catabolism [54,55]. However, this was not supported by the result of total proteins. Once again, it is observed that TTI, when contained in the nanoformulation, seems to contribute positively to specific parameters compared to CW, which seems to impair renal homeostasis due to the absence of TTI.

#### 2.2.5. Assessing Liver Function

For liver enzymes (Table 1), it was noted that for ALT and GGT, there were no significant differences between groups (*p* > 0.05). However, the concentration of AST showed a significant reduction (*p* = 0.0339) in the group treated with ECW compared to the group without treatment. This same finding was demonstrated in the study by Costa et al. [29]. AST showed a significant reduction (*p* = 0.05) in the groups treated with a standard diet, ECW, and CW compared to the group without treatment, indicating that all three treatments improve the concentration of this enzyme, following the concentrations presented in eutrophic animals (Table 2). The reduction of AST in the group treated with ECW reinforces the beneficial effects on the liver. This enzyme is increased in expansive lesions in the liver, such as hepatocellular carcinoma, viral hepatitis, and cirrhosis [56]. Thus, these results evidence that ECW could be associated with a possible beneficial effect of liver damage and reduced IR because the animals consumed the HGLI diet, related to hyperglycemia and, consequently, hepatic lipogenesis [19,20].

Together with other data, the liver enzyme evaluation is essential for recognizing patients affected with NAFLD, as these usually do not show signs or symptoms. The disease hypothesis is confirmed when patients have elevated liver enzymes [12]. However, this assessment alone does not support the presence of the disease. Therefore, the inclusion of other non-invasive methods is necessary. In specific cases, liver biopsy is performed because it is expensive, invasive, and subject to sampling errors [12,14,57,58].

### 2.3. Liver Damage Assessment Scores

**Figure 2:**
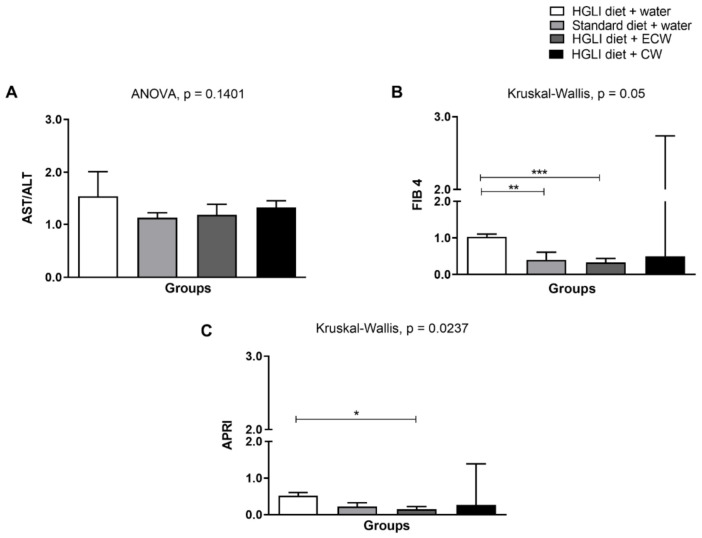
Assessment of liver damage scores. (**A**) AST/ALT ratio. (**B**) FIB-4. (**C**) APRI. Experimental groups: (1) HGLI diet + water; (2) Standard diet + water; (3) HGLI diet + ECW; (4) HGLI diet + CW. Values were expressed as mean and standard deviation. The Kolmogorov-Smirnov test was used to evaluate the normality of the data. AST/ALT ratio presented parametric distribution, so the ANOVA test with Tukey’s post-hoc test was used to determine the significant differences.FIB-4 and APRI showed non-parametric distribution, so the Kruskal-Wallis test and Dunn’s post-test were used to detect significant differences. * Values were significantly different for APRI score, according to Kruskal-Wallis and Dunn’s post-hoc test (*p* < 0.05). ** Values were significantly different for FIB 4 score, according to Kruskal-Wallis and Dunn’s post-hoc test (*p* < 0.001). *** Values were significantly different for FIB 4 score, according to Kruskal-Wallis and Dunn’s post-hoc test (*p* < 0.0001). Mean and standard deviation. HGLI: high glycemic index and glycemic load diet; AST: oxalacetic glutamic transaminase; ALT: alanine transaminase; FIB-4: Fibrosis-4 Index for Liver Fibrosis; APRI: AST to Platelet Ratio Index; ECW trypsin inhibitor isolated from tamarind seeds encapsulated with whey protein isolate and chitosan (1:2:2 *w/w/w*); CW: chitosan–whey protein isolate nanoparticles (2:2 *w*/*w*).

Non-invasive methods, such as AST/ALT ratio, FIB-4, and APRI, were used to evaluate liver damage (Figure 2). However, it is essential to mention that no results were found in the literature on assessing these scores in experimental designs similar to those applied in the present study, mainly using nanoformulations.

For the AST/ALT ratio (Figure 2A), there was no significant difference between the groups evaluated (*p* > 0.05), even so, all groups obtained an average of AST/ALT ratio >1, which is indicative of fibrosis accentuated and progression of the disease when it has values greater than 1 [59,60]. The high-carbohydrate diet used in this study may have been the main factor for this finding since the condition of fibrosis is aggravated by the high carbohydrate content of the diet. In addition, the HGLI diet consumption increased lipogenesis again, besides adipose tissue expansion with subsequent proinflammatory cytokine production and the recruitment of immune system cells that enable hepatic fibrogenesis [8,61,62,63].

Even though the AST/ALT ratio is widely known to be a good NAFLD marker, a study conducted with patients with NAFLD showed that this parameter was the least likely between APRI and FIB-4 scores to indicate a difference between mild to moderate and advanced fibrosis [63]. Therefore, APRI and FIB-4 scores can be used to follow-up on patients with early NAFLD along with the AST/ALT ratio when the liver biopsy does not show a clear indication.

FIB-4 and APRI are also non-invasive methods that estimate fibrosis in the liver and are generally used for patients with hepatitis C but are being used as adjuvants to NAFLD diagnosis, in addition to imaging tests and biochemical parameters [14]. The analysis of FIB-4 (Figure 2B) showed a significant difference (*p* = 0.05) between animals without treatment and those treated with a standard diet and with ECW. A literature review showed that FIB-4 is a valuable screening tool to be applied routinely in clinical practice since it can accurately exclude patients with advanced fibrosis [64].

**Figure 3:**
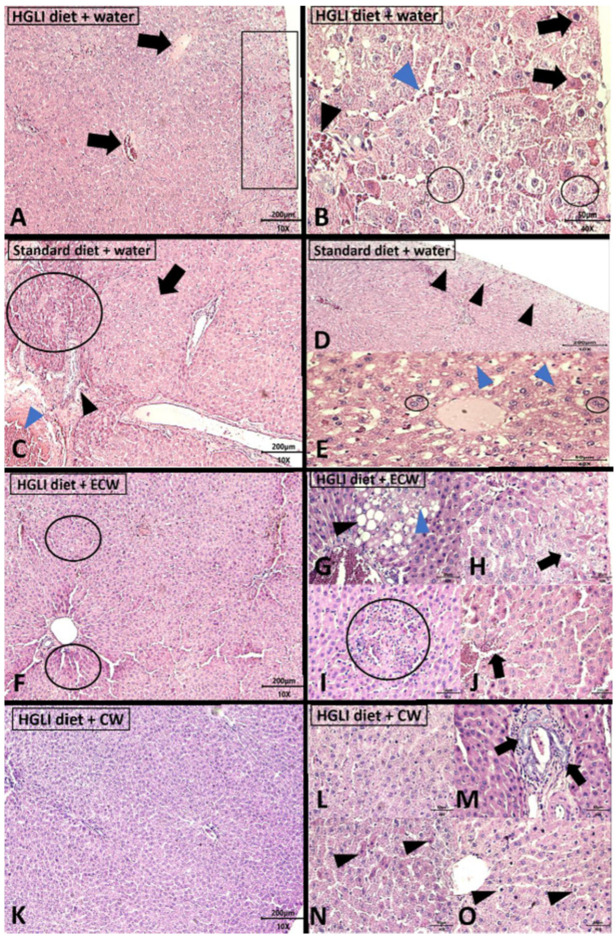
Histopathological analysis Wistar rats livers stained with hematoxylin and eosin (H,E). (**A,B**): HGLI diet + water (*n* = 5); (**C**–**E**): standard diet + water (*n* = 5); (**F**–**J**): HGLI diet + ECW (*n* = 5); (**K**–**O**): HGLI diet + CW. (**A**): Panoramic evidence of the liver, with the presence of dilated blood vessels due to hyperemia (black arrow), in addition to an area of ballooning degeneration and hepatocytes suggestive of necrosis (black rectangle) in the peripheral organ portion (H,E-10× objective). (**B**): Presence of hepatocytes suggestive of necrosis (black arrows); hepatocytes showing ballooning degeneration (black circles); evidence of sinusoidal capillaries dilatation (blue arrowhead); hyperemia in the centrilobular vein (black arrowhead) (H,E-40× objective). (**C**): extensive area of hepatocytes suggestive of necrosis (black circle); evidence of pseudolobule (black arrow); bile ductal proliferation (black arrowhead); intense hyperemia (blue arrowhead) (H,E-10× objective). (**D**): an external band of ballooning degeneration (black arrowheads) (H,E-10× objective). (**E**): the presence of binucleated hepatocytes (black circle) and microvesicular steatosis (blue arrowhead) (H,E-40× objective). (**F**): liver panoramic view, showing few areas of necrosis (black circles) (H,E-10× objective). (**G**): evidence of a steatosis focal area, showing microvesicular steatosis (blue arrowhead) and macrovesicular steatosis (black arrowhead) (40× H,E-Objective). (**H**): area of ballooning hepatocytes degeneration (black arrow) (H,E-40× objective). (**I**): focal area of mononuclear inflammatory infiltrate (black circle) (H,E-10× objective). (**J**): hyperemia and dilation of sinusoidal capillaries (black arrow) (H,E-10× objective). (**K**): panoramic view of the liver showing the predominant maintenance of the normal architecture (H,E-10× objective). (**L**): evidence of hepatic cord morphology maintenance, without evidence of hyperemia (H,E-40× objective). (**M**): the presence of bile duct proliferation (black arrows) (H,E-40× objective). (**N**): scarce hepatocyte necrosis (black arrowhead) (H,E-40× objective). (**O**): scarce hepatocyte ballooning degeneration (black arrowhead) (H,E-40× objective). H,E: hematoxylin and eosin. HGLI: diet with a high glycemic index and high glycemic load. ECW trypsin inhibitor isolated from tamarind seeds encapsulated with whey protein isolate and chitosan (1:2:2 *w/w/w*); CW: chitosan–whey protein isolate nanoparticles (2:2 *w*/*w*).

In evaluating the APRI score (Figure 3C), there was a significant difference (*p* = 0.0237) between the group without treatment and treated with ECW, showing that it has a better aspect related to hepatic fibrosis. Thus, if there is a joint dietary change, this improvement could be enhanced. In addition, Kolhe et al. [65] showed that APRI is an accurate method for ruling out significant fibrosis patients with NAFLD.

It is noteworthy that combined models (routine clinical variables based on blood markers and non-invasive scores) that reflect the dynamic nature of the fibrogenic process appear to have greater diagnostic precision and predictive value in the advanced fibrosis identification in NAFLD patients [12,64]. Thus, together with the significant reduction of AST and ALP in the group treated with ECW, these results reinforce the evidence that this nanoformulation could be associated with a better aspect related to liver damage and improvement in fibrosis.

### 2.4. Histopathological Analysis of Liver Tissue

The liver is one of the best physiological and pathological status markers in studies with humans and animals. In addition, it is essential in the metabolism of carbohydrates and lipids, and its morphology allows the observation and identification of several liver diseases [12], mainly NAFLD. Among the four animal groups evaluated, acute hepatitis was the alteration found in all liver tissues (Figure 3).

Wistar rat livers in the group that received an HGLI diet and water presented liver parenchyma alterations (Figure 3A,B). All animals in this group had hyperemia and microvesicular steatosis. In addition, most animals had subcapsular ballooning degeneration (Figure 3A) and areas suggestive of necrosis (Figure 3A,B). Microvesicular steatosis is generally associated with severe liver dysfunction related to changes in the beta-oxidation pathway of free fatty acids [66]. In this group, half of the animals showed hepatocytes degeneration with a granular aspect inside some hepatic lobules and sinusoid capillaries dilation (Figure 3B).

The changes presented by the untreated animals are due to the effect resulting from the HGLI diet ingestion for 17 weeks, which occurred before the beginning of the treatments. Furthermore, this was also observed in the study conducted by Luz et al. [20], who used the HGLI diet and found adipocyte infiltration in the liver, characterizing macrovesicular steatosis in animals. This same diet had already caused dyslipidemia in another study [19]. Pompili et al. [67] also demonstrated in a NAFLD/NAHNA model using mice that long-term carbohydrate-rich diet use was detrimental to the development and progression of liver damage. In addition, Pierce et al. [68] have already shown that diet-induced liver damage correlates positively with liver lipogenesis and fat content with no feedback inhibition of hepatic steatosis.

The group of animals that consumed the HGLI diet for 17 weeks and did not receive treatment during the experiment showed a significant increase (*p* < 0.05) in AST and ALP. In addition, although not significant, an increase in ALT average concentrations corroborated the liver damage confirmed by histopathology. Donnelly et al. [69] investigated patients with NAFLD, infused and fed orally with stable isotopes for four days, to mark and screen free fatty acids before the analyzed liver biopsy. The researchers observed that the entry of fat into the liver occurred from three sources: most of the liver fat (59%) originated from lipolysis of adipocytes; the second significant contribution to liver fat in NAFLD patients was related to carbohydrate intake via lipogenesis again with 26%; and the third-largest proportion through dietary fat (15%), showing once again that lipo- and gluco-toxicity play a central role in NAFLD. This evidence supports the findings obtained in the present study for the untreated group due to histopathology results and significantly elevated fasting blood glucose (*p* < 0.05) compared to the other groups.

Regarding the livers of the Wistar rats in the group that received a standard diet and water (Figure 3C–E), it was observed that all had microvesicular steatosis (Figure 3E) and ballooning degeneration (Figure 3D). However, this was less evident than in the animals of the group without treatment. Some animals even showed hyperemia (Figure 3C), scarce areas of inflammatory infiltrate in some portal spaces, pseudolobule formation area (Figure 3C), binucleated hepatocytes (Figure 3E), and some areas suggestive of necrosis (Figure 3C), but to a lesser extent degree than the untreated group. Only one animal showed indicative bile duct proliferation (Figure 3C).

These findings indicate that treatment with a standard diet was beneficial for the histopathological parameters of the liver, resulting from the HGLI diet, which has confirmed harmful effects on the liver [20]. Another result that can reinforce these histological findings is that in the group treated with a standard diet, there was a significant reduction in the value of FIB-4 when compared with the group without treatment, showing a better aspect of severe fibrosis [64].

For the group treated with the HGLI diet and ECW (Figure 3F–J), it was necessary to carry out a more robust assessment of all the effects of nanoformulation on the liver. As a result, few indicative areas of necrosis were observed (Figure 3F). In one animal, dilatation of sinusoidal capillaries (Figure 3J) and hyperemia (Figure 3J) was more discreet than in animals without treatment and treated with a standard diet. There was also slight ballooning degeneration (Figure 3H) and mononuclear inflammatory infiltrates (Figure 3I). Only one animal presented micro and macrovesicular steatosis (Figure 3G) in zone 3 of the hepatic acini. These changes were close to the central lobular vein, which is the site that receives the least supply of oxygen and nutrients. In addition, microvesicular steatosis was less evident than in groups without treatment and treated with a standard diet (Figure 3E).

Macrovesicular steatosis is a less severe lesion than microvesicular steatosis and was observed in groups of animals without treatment and treated with a standard diet. Some authors suggest that the fatty liver, at the expense of macrovesicular steatosis, is more resistant to possible other aggressions. In contrast to microvesicular steatosis [70], which represents a more severe condition, with a risk of death from liver failure [71], making the liver more sensitive to secondary deleterious mechanisms [72]. One study showed that steatosis and alterations related to NAFLD in adults were prevalent in the hepatic acin zone 3 [73], receiving a lower supply of oxygen and nutrients [74]. The group treated with ECW compared to those without treatment and treated with a standard diet showed benefits in organ morphology. It is known that the harmful changes are due to the HGLI diet ingestion for 17 weeks, which has already proven to be detrimental to the liver [20].

Probably, the 10-day treatment period was not enough to observe sustained morphological effects. However, concerning the biochemical parameters of hepatic involvement, the concentrations of AST and ALP for the group treated with ECW were significantly reduced (*p* < 0.05) compared to the group without treatment. In addition, the reduction in ALP and the values of fibrosis assessment scores (FIB-4 and APRI) were observed only in the group treated with ECW compared to those without treatment (*p* < 0.05), corroborating the attenuation of marked fibrosis [66]. Another fact that supports the potentialized bioactivity of the TTI contained in ECW concerning the other treatments, including compared to the conventional treatment (standard diet), is a nutritionally adequate diet.

Carvalho et al. [26] evaluated Wistar rats diagnosed with MS induced by the HGLI diet and treated with pure non-encapsulated TTI. The researchers did not observe the AST reduction being a novelty for nanoencapsulated TTI. Therefore, the result suggests that ECW may be an adjuvant molecule in the NAFLD treatment, mainly if associated with the dietary change proposed by Costa et al. [29].

In the group treated with CW (Figure 3K–O), hyperemia was observed in all animals, also noticed was areas of bile duct proliferation (Figure 3M) and ballooning degeneration (Figure 3N). In most animals, there were areas suggestive of necrosis on the organ periphery and inside the liver lobules (Figure 3N). In one animal, preserved morphology was observed towards the center of the hepatic parenchyma (Figure 3K,L). Despite maintenance of typical architecture in this animal, it is already known that CW is reactive to other organs, as evidenced by Matias et al. [30].

A finding only demonstrated in animals treated with CW and those treated with a standard diet was bile duct proliferation (Figure 3M), which may indicate intrahepatic cholestasis (IHC) with possible biliary atresia and fibrosis progression. The liver fibrosis progression can be rapid and aggressive in these cases. This proliferation can also be stimulated by inflammatory factors causing loss or damage to the liver tissue [75,76,77]. In the literature, the association between intrahepatic cholestasis (IHC) and pre-existing NAFLD remains poorly investigated, with no prospective studies, mainly under the conditions of the present study using nanoparticles.

Data suggestive of the specific relationship between NAFLD and the risk of cholestasis are scarce in the literature. However, a case-control study carried out with pregnant women by Monrose et al. [78] was published as a summary in Digestive Disease Week 2019. In that cohort, patients with cholestasis were significantly more likely to be diagnosed NAFLD than controls. A letter to the editor published (2021) in the Journal of Hepatology highlights the need to clarify the association between IHC and NAFLD, in addition to the scarcity of effective therapies and the increasing prevalence of NAFLD worldwide [79]. The suggestive areas of necrosis reveal this inflammation condition and fibrosis in the group treated with CW and ballooning degeneration observed in histological sections and not significantly reducing any non-invasive scores that evaluate the best aspect of fibrosis in the tissue hepatic.

Thus, ECW had already been presented as a hypoglycemic molecule [30] and a possible hepatoprotective effect by reducing AST [29]. However, by assessing the impact on liver status more broadly, the findings for ECW were confirmed in the present study. In addition, there was an unprecedented reduction in the concentration of ALP in animals treated with ECW and a significant decrease in the non-invasive scores for the assessment of liver fibrosis APRI and FIB-4. The latter is similar to the effect of a nutritionally adequate diet. In the histopathological analysis of the liver, the findings mentioned above are confirmed for the animals that received ECW, which also showed a better aspect of the liver parenchyma than animals without treatment, mainly in reducing necrosis hyperemia, and ballooning degeneration.

These occurrences in the liver state of Wistar rats were evidenced with these animals in a stage of life equivalent to the human age of 20 years, according to Andreollo et al. [80]. This reinforces that the behavioral factors, mainly related to high glycemic index and glycemic load diet, are crucial for liver diseases such as NAFLD [8,64], even for young adults.

In this study, the CW proved to be an extremely reactive nanoformulation, as shown in the results mentioned above [30], which was not observed for the ECW, evidencing this nanoparticle’s beneficial effect with ITT. In a previous study, Queiroz et al. [28] pointed out that the whey protein isolate, when interacting with chitosan in the preparation of the solution, at pH 5.5, to promote the encapsulation process, changes the protonation state resulting in a negative surface charge evidenced in ECW by Zeta Potential. Thus, chitosan predominantly interacts with TTI, stabilizing the system through molecular interactions such as Van der Waals forces, hydrophobic and hydrogen bonds, and interacting with some anionic regions of the whey protein isolate [33,81,82]. Then, believed through ECW, the chemical interactions of TTI with chitosan reduced its reactivity [30].

Thus, Costa et al. [29] showed that the encapsulating agent present on the surface of the ECW is most likely the protein isolated from whey, which undergoes denaturation at acidic pH. In addition, Matias et al. [30], simulating the gastrointestinal digestion of ECW, proved through High-Performance Liquid Chromatography that, possibly in the intestine, there is the presence of only ECW without whey protein isolate. Since after digestion, the protein peaks characteristic of this protein disappears in the gastric and intestinal phases, showing that only chitosan interacts with TTI in the intestine. This evidence corroborates that, once administered orally, there is no risk of absorption of CW-type nanoparticles resulting from the digestion of ECW.

Studies investigating how ECW absorption occurs have not yet been conducted. Thus, this is an aspect to be explored in future research. However, the several studies already published with significant systemic or morphological effects and of the gene expression, in preclinical studies with animals treated with unencapsulated TTI [23,24,25,26,27] and ECW (encapsulated TTI) [29,30] compared with untreated animals are evidence that attests to its absorption.

## 3. Materials and Methods

### 3.1. Obtaining the TTI

Tamarind (*Tamarindus indica* L.) was obtained and identified botanically by the Brazilian Institute for the Environment and Renewable Natural Resources (Ibama) in a seed bank located in Natal/RN (Brazil). It was registered in the National Genetic Heritage Management System and Associated Traditional Knowledge (SisGen) under AF6CE9C.

The ITT was obtained from the cotyledon flour of tamarind seeds after following the steps of protein extraction (50mM Tris-HCl buffer, pH 7.5), fractionation with ammonium sulfate in three saturation ranges (0–30%, 30–60%, and 60–90%), and isolation by affinity chromatography on Trypsin-Sepharose CNBr 4B (elution with 5mM HCl at a flow of 0.5 mL/min), according to Carvalho et al. [24].

All reagents, substrates, solutions, and kits used in the research were of a high degree of purity and obtained from Sigma-Aldrich^®^ (St. Louis, MO, USA) and VETEC Química Fina Ltd.a^®^, (Rio de Janeiro, Brazil) and Merck^®^ (Rio de Janeiro, Brazil).

To quantify the total protein content the Bradford method was used [83]. Trypsin inhibition assays were performed using 10 μL (3 μg) aliquots of trypsin and 1.25 Mm BApNa (Nbenzoyl-dl-arginine-p-nitroanilide) as a substrate, as proposed by Kakade [84]. The antitrypsin activity was expressed in percentage (%) and specific activity in IU/mg of protein. The purity degree and molecular mass of the protein isolate were evaluated by electrophoretic analysis on 12.5% SDS-PAGE, according to Laemmli [85]. The proteins were revealed according to Oakley et al. [86], using silver nitrate. An Amersham™ ECL™ Rainbow™ Marker—Full range GE Healthcare molecular weight marker (225, 150, 102, 76, 52, 38, 31, 24, 17, and 12 kDa) was used to estimate the isolated protein molecular mass.

### 3.2. Production and Characterization of Nanoformulations

The nanoformulations were obtained in triplicate using the nanoprecipitation technique, according to Luque-Alcaraz et al. [87], based on modifications proposed by Queiroz et al. [28] and Matias et al. [30]. Chitosan of low molecular weight (approximately 50,000–190,000 daltons) and a degree of deacetylation around 75–85% was purchased from Sigma-Aldrich^®^ (St. Louis, MO, USA) and subjected to purification based on Kumari et al. [88] with changes proposed by Queiroz et al. [28]. Alibra Ingredientes Ltd.a^®^ (Parque Industrial—M.C. Rondom/PR) supplied the whey protein isolate.

A 1:2:2 (*w/w/w*) ratio was used to obtain TTI nanoparticles, chitosan, and whey protein isolate (ECW), respectively, and a 1:1 (*w*/*w*) without TTI (CW). After freeze-drying, the powdered nanoformulations were characterized according to Matias et al. [30]. Then, ECW and CW were dispersed in acetone and dripped onto silicon plates for morphology evaluation by Scanning Electron Microscopy (SEM). For diameter evaluation by Laser diffraction, the nanoformulations were dispersed in water and previous crosslinking using formaldehyde. Furthermore, ECW and CW were characterized as to the chemical interactions present in the systems obtained by Fourier Transform Infrared Spectroscopy (FTIR), being previously homogenized with potassium bromide.

To evaluate the incorporation of TTI in the particles, first, it was necessary to measure the antitrypsin activity. As it is a trypsin inhibitor, the wavelengths of maximum absorption used for measurement are similar to those of encapsulating agents. Based on this, the amount of TTI present in the particles was determined by measuring their antitrypsin activity as proposed by Kakade [84], as already established by Matias et al. [30]. A total 3.5 mg of ECW was used to ensure 100% antitrypsin activity as described by Queiroz et al. (2018). The encapsulation efficiency (%) was initially made by solubilizing the ECW and CW separately, in water, vortexed for 1 min, and centrifuged (56,000× *g*/5 min at 37 °C), the supernatant excluded, and the antitrypsin assay was performed. The test was performed in triplicate. Thus, after obtaining these results, the incorporation efficiency was determined, according to Kumari et al. [88], based on the formula below:

Encapsulation efficiency (%) = (inhibitor in particles/total inhibitor used) × 100(1)

### 3.3. Study Design

**Figure 4:**
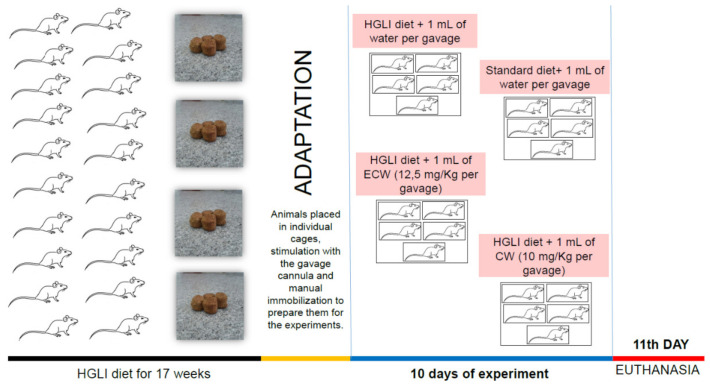
Experimental design summary. HGLI: high glycemic index and glycemic load diet; ECW trypsin inhibitor isolated from tamarind seeds encapsulated with whey protein isolate and chitosan (1:2:2 *w/w/w*); CW: chitosan–whey protein isolate nanoparticles (2:2 *w*/*w*).

The effect of nanoformulations on liver status was evaluated in adult male Wistar rats (*Rattus norvegicus albinus*) (*n* = 20) fed with a high glycemic load and high glycemic index diet (HGLI diet) for 17 weeks *ad libitum* (Figure 4). Initially, the 20 animals were adapted for five days, consuming the same HGLI diet, allocated in individual cages, submitted to stimulation of the gavage, and manual immobilization to adapt them for the experiments.

After the adaptation, the animals were randomly allocated to four groups to start the ten days of investigation: (1) HGLI diet + 1 mL of water per gavage (*n* = 5); (2) standard diet (Labina^®^) + 1 mL of water per gavage (*n* = 5); (3) HGLI diet + 1 mL of ECW dispersion (12.5 mg/kg per gavage) (*n* = 5); and (4) HGLI diet + 1 mL of CW dispersion (10.0 mg/kg per gavage) (*n* = 5). The animals received the treatment once a day. They were kept in individual cages, with water and feed offered ad libitum, under 12 h light/dark cycle conditions, with an average temperature of 23–25 °C and controlled humidity (50 ± 5%).

Thus, the diets used in the experimental treatment were the standard Labina^®^ diet (Paulínia, São Paulo, Brazil), considered nutritionally adequate, and the HGLI diet with a high glycemic index (77.6), high glycemic load (38.8), and a percentage of macronutrient distribution of 64% of carbohydrates, 19.3% of proteins, and lipids of 16.7% [20].

It should be emphasized that the safe concentration used for ECW (12.5 mg/kg) and CW (10.0 mg/kg) was established according to Costa et al. [29]. Furthermore, the study was approved by the Ethics Committee on the Use of Animals (CEUA-UNP, Natal/RN) under protocol No. 019/2017 and was carried out following the Guide for the Care and Use of Laboratory Animals [89].

On the eleventh day of the experiment, the animals were euthanized using Zoletil^®^ 50 (50–80 mg/mL). Blood collection was performed by cardiac puncture to analyze the following hematological and biochemical parameters: platelets, hemoglobin, hematocrit, total leukocyte count, AST, glutamic-pyruvic transaminase (ALT), gamma-glutamyltransferase (GGT), alkaline phosphatase (ALP), triglycerides, total cholesterol, high-density lipoprotein (HDL-c), VLDL-c, low-density lipoprotein (LDL-c), albumin, fasting glucose, insulin, HOMA-IR, HOMA-β, urea, and creatinine. In addition, the livers were collected and stored in a formaldehyde solution (4%) for later histopathological analysis.

### 3.4. Evaluation of Hematological and Biochemical Parameters

The method used to evaluate hematological and biochemical parameters was the automated enzymatic colorimetric (Labtest^®^, Natal, RN, Brazil). For HDL-c analysis, the value was calculated by an indirect method, according to the Friedewald equation. For insulin dosage, was used the Rat/Mouse Insulin Elisa kit (Millipore EZRMI13K).

The homeostasis assessment model (homeostasis model assessment of insulin resistance and β cells–HOMA–IR and HOMA β, respectively) were used to measure the IR and the functionality of pancreatic β cells. The determinations were made based on the formulas described below, proposed by Matthews et al. [90]:

HOMA-IR = fasting insulin (µUImL) × fasting glucose (mmol/L)/22.5(2)

HOMA β = (20 × μUI/mL insulin)/(Glucose mmol/L − 3.5)(3)

Reference values were considered for the hematological and biochemical parameters evaluated in male, adult, eutrophic Wistar (320–380 g), acclimated under the same experimental conditions in question, consuming a standard Labina^®^ diet (Table 2). All values were following Matias et al. [30] and Costa et al. [29].

### 3.5. Liver Damage Assessment Scores

For liver damage and fibrosis, non-invasive score assessments were used: AST/ALT ratio, FIB-4, and APRI. The FIB-4 was calculated as proposed by Sterling et al. [91], using age, AST, ALT, and platelet concentration, according to the formula 1:

FIB-4 = [{age (years) × AST (U/L)}/ platelet count (10^9^/L) × {ALT (U/L) 1/2}] (4)

According to Wai et al. [92], the APRI was calculated and used the AST concentration data, the upper limit of normality of AST, and the platelet count. Therefore, the formula 2 used was APRI:

APRI= [(AST value/upper limit of normal AST) × 100]/platelet count {10^9^/L} (5)

These scores were evaluated for all animals in the four groups studied. The age of the animals was 31 weeks, which is equivalent to a human age of approximately 20 years [80]. Concerning the upper limit of normality of AST, the value of 67 IU/L was used. It was considered the maximum value of AST obtained in the same experimental conditions, attributed to the calculation of APRI for all animals.

### 3.6. Histopathological Analysis of Liver Tissue

The livers were collected through a longitudinal cut, with the aid of scissors, from the base of the abdomen to the entire segment of the external area, showing the whole abdominal and thoracic cavity for later histological analysis. The organs were fixed in 10% formaldehyde. The liver tissues were individually packed, in plastic pots with lids, cleaved, and the fixed material was dehydrated in an increasing alcoholic series (70%, 80%, and 90%), diaphanized in xylol, impregnated, and embedded in histological paraffin. Subsequently, the fragment was sectioned in a microtome (Leica RM2235, Buffalo Grove, IL, USA) at 5 μm. The sections were stretched on a glass slide, stained with routine staining (hematoxylin and eosin), covered with a coverslip, and, finally, were analyzed under a light microscope. The histological slides obtained came from the left lobe of the liver (four slides with three sections per slide).

For microscopic analysis performed by a pathologist, the slides obtained for the different experimental groups were evaluated for organ presentation, sufficient quantity of specimen on the slide, and good section quality. We opted for the detailed histopathological description of the liver. The examiner was blind to the groups and evaluated the presence of alterations indicative of damage, emphasis on the histological organization of the organ, and the presence of fibrosis/necrosis and/or infiltration of fat and inflammatory cells with the aid of a microscope B-800 (Optika microscopes, Ponteranica, Bergamo, Italy) with 4×, 10× and 40× objectives.

### 3.7. Statistical Analysis

According to the Cochran model [93], the sample size was calculated, considering a simple and random sampling, according to the 3Rs principle (reduction, refinement, and substitution). An anticipated variation coefficient of 10% was adopted with an error probability of less than 5% and a power of 90%, corresponding to 4.4 animals, that is, five animals per study group. Thus, a physiologically significant parameter difference was evaluated when the treatment affected greater than or equal to 25%.

The Kolmogorov-Smirnov test was applied to evaluate the normality of the data. Thus, in the non-parametric variables, the Kruskal-Wallis test and Dunn’s post-test were used to verify the difference between the groups studied. For parametric data, ANOVA with Tukey’s post-test was used to determine the significant differences. In both cases, *p* values ≤ 0.05 were considered statistically significant. Values were expressed as mean and standard deviation. Equal letters indicate that there is no significant difference between the groups evaluated for each parameter. GraphPad Prism software, version 5.0 (Graph Pad Software, San Diego, CA, USA), was used for the statistical analysis of the data obtained.

## 4. Conclusions

ECW proved to be a molecule with great potential given the data obtained. TTI present in the ECW nanoparticle promoted bioactive effects not presented by CW. ECW improved biochemical parameters, non-invasive scores, and histopathological analyses related to the general state of the liver of Wistar rats fed a high glycemic index diet and high glycemic load. It should also be noted that the association of the assessments performed in this study is unprecedentedly related to assessing non-invasive liver damage scores, together with histopathological analysis in an animal model fed with an HGLI diet and treated with nanoparticles containing TTI. Thus, given the results presented, ECW proved to be a candidate for clinical studies to be a possible adjuvant, along with diet therapy treatment, in NAFLD therapy and its progression.

## Figures and Tables

**Table 1:** Hematological parameters, fasting glucose, insulin, HOMA-IR, HOMA-β, lipid profile, assessing renal and liver function in adult male Wistar rats fed with the HGLI diet acclimated in the UnP/bioterium Brazil in January 2019.

Parameters	HGLI Diet + Water	Standard Diet + Water	HGLI Diet + ECW	HGLI Diet + CW
Hemoglobin (g/dL)	13.34 (0.77) ^a^	12.94 (0.46) ^a^	13.56 (1.29) ^a^	14.73 (3.17) ^a^
Hematocrit (%)	39.62 (2.31) ^a^	39.14 (3.47) ^a^	32.90(15.61) ^a^	44.18 (9.52) ^a^
Leukocyte total count (×10^3^/µL)	7.78 (0.57) ^a^	7.34 (0.97) ^a^	7.34 (0.69) ^a^	4.91 (1.06) ^b^
Platelets (×105 /µL)	2.45 (0.43) ^a^	3.95 (1.20) ^a^	4.38 (0.65) ^a^	4.23 (2.86) ^a^
Fasting blood glucose (mg/dL)	175.94 (21.07) ^a^	141.73 (2.81) ^b^	146.28 (7.44) ^b^	149.13 (6.97) ^b^
Insulin (µU/mL)	4.58 (0.64) ^a^	4.08 (0.55) ^a^	4.44 (0.45) ^a^	19.41 (1.64) ^b^
HOMA-IR	2.01 (0.49) ^a^	1.46 (0.25) ^a^	1.60 (0.15) ^a^	7.16 (0.90) ^b^
HOMA-BETA	14.80 (1.87) ^a^	18.00 (1,93) ^a^	19.39 (3.02) ^a^	81.30 (3.23) ^b^
Total cholesterol (mg/dL)	87.21 (17.68) ^a^	82.57 (5.57) ^a^	82.32 (6.22) ^a^	64.11 (6.63) ^b^
HDL-c (mg/dL)	30.20 (5.11) ^a^	32.86 (2,60) ^a^	36.72 (3.41) ^a^	22.83 (3.02) ^b^
LDL-c (mg/dL)	14.45 (5.15) ^a^	16.00 (1.13) ^a^	16.79 (0.90) ^a^	21.11 (7.93) ^a^
VLDL-c (mg/dL)	41.19 (21.83) ^a^	34.28 (5.91) ^a^	28.77 (9.00) ^a^	20.18 (7.14) ^a^
Triglycerides (mg/dL)	72.24 (25.76) ^a^	79.98 (5.67) ^a^	83.96 (4.51) ^a^	105.58 (39.66) ^a^
Total protein (mg/dL)	6.00 (0.46) ^a^	6.24 (0.33) ^a^	5.98 (0.60) ^a^	6.36 (0.32) ^a^
Albumin (mg/dL)	3.89 (0.23) ^a^	3.50 (0.43) ^a^	3.82 (0.26) ^a^	2.42 (0.26) ^b^
Urea (mg/dL)	26.47 (1.16) ^a^	25.64 (1.66) ^a^	26.48 (1.72) ^a^	36.34 (7.38) ^b^
Creatinine (mg/dL)	0.65 (0.10) ^a^	0.70 (0.10) ^a^	0.88 (0.19) ^a^	0.60 (0.22) ^a^
AST (U/L)	83.77 (25.75) ^a^	57.62(17.41) ^a,b^	51.16 (7.57) ^b^	72.05 (6.48) ^a,b^
ALT (U/L)	60.26 (32.90) ^a^	51.02 (14.73) ^a^	44.64(12.01) ^a^	54.78 (6.56) ^a^
GGT (U/L)	31.28 (6.16) ^a^	36.82 (5.97) ^a^	30.30(15.47) ^a^	22.50 (3.87) ^a^
ALP (U/L)	80.54 (13.44) ^a^	62.00 (9.53) ^b^	61.20(10.76) ^b^	60.35 (6.70) ^b^

Experimental groups: (1) HGLI diet + water; (2) Standard diet + water; (3) HGLI diet + ECW; (4) HGLI diet + CW. All the values were expressed as mean and standard deviation. Equal letters indicate that there is no significant difference between the groups evaluated for each parameter. The Kolmogorov-Smirnov test was applied to evaluate the normality of the data. Hematocrit, platelets, AST, ALP, and total cholesterol data showed non-parametric distribution, so the Kruskal-Wallis test and Dunn’s post-test were used to detect significant differences. The other variables showed parametric distribution. Therefore, an ANOVA test with Tukey’s post-hoc test was used to determine the significant differences. Equal lower case letters, in the same row: means did not differ significantly according to the Tuckey’s post-hoc test, *p* values ≤ 0.05. HGLI: high glycemic index and high glycemic load diet; AST: aspartate aminotransferase; ALT: alanine transaminase; GGT: gamma-glutamyl transferase; ALP: alkaline phosphatase; HDL-c: high-density lipoprotein; LDL-c: low-density lipoprotein; VLDL-c: very-low-density lipoprotein; HOMA-IR: Homeostasis Evaluation Model for Insulin Resistance; HOMA-β: Model for Assessment of β Cell Homeostasis; ECW trypsin inhibitor isolated from tamarind seeds encapsulated with whey protein isolate and chitosan (1:2:2 *w/w/w*); CW: chitosan–whey protein isolate nanoparticles (2:2 *w*/*w*).

**Table 2:** Reference values of blood parameters in male, adult, eutrophic Wistar rats acclimated in the bioterium of UnP/Brazil in January 2019.

Parameters	Mean (SD)
Hemoglobin (g/dL)	23.90 (15.70)
Hematocrit (%)	39.80 (4.82)
Leukocyte total count (×10^3^/µL)	6.44 (0.66)
Platelets (×105/µL)	3.41 (0.69)
Fasting blood glucose (mg/dL)	88.80 (17.87)
Insulin (µU/mL)	12.17 (0.73)
HOMA-IR	2.67 (0.63)
HOMA-BETA	47.44 (9.07)
Total cholesterol (mg/dL)	112.00 (54.00)
HDL-c (mg/dL)	23.40 (4.04)
LDL-c (mg/dL)	22.76 (4.05)
VLDL-c (mg/dL)	20.65 (5.59)
Triglycerides (mg/dL)	100.18 (29.80)
Total protein (mg/dL)	6.46 (0.42)
Albumin (mg/dL)	2.30 (0.26)
Urea (mg/dL)	32.20 (7.08)
Creatinine (mg/dL)	0.80 (0.16)
AST (U/L)	49.20 (11.90)
ALT (U/L)	43.40 (6.11)
GGT (U/L)	33.50 (3.38)
ALP (U/L)	64.50 (6.59)

Values were expressed as mean and standard deviation. AST: aspartate aminotransferase; ALT: alanine transaminase; GGT: gamma-glutamyl transferase; ALP: alkaline phosphatase; HDL-c: high-density lipoprotein; LDL-c: low-density lipoprotein; VLDL-c: very-low-density lipoprotein; HOMA-IR: a model for assessing insulin resistance homeostasis; HOMA-beta: a model for the evaluation of β cell homeostasis.
